# GoldenBraid: An Iterative Cloning System for Standardized Assembly of Reusable Genetic Modules

**DOI:** 10.1371/journal.pone.0021622

**Published:** 2011-07-07

**Authors:** Alejandro Sarrion-Perdigones, Erica Elvira Falconi, Sara I. Zandalinas, Paloma Juárez, Asun Fernández-del-Carmen, Antonio Granell, Diego Orzaez

**Affiliations:** Instituto de Biología Molecular y Celular de Plantas (IBMCP), Consejo Superior de Investigaciones Científicas (CSIC), Universidad Politécnica de Valencia (UPV), Valencia, Spain; Virginia Tech, United States of America

## Abstract

Synthetic Biology requires efficient and versatile DNA assembly systems to facilitate the building of new genetic modules/pathways from basic DNA parts in a standardized way. Here we present GoldenBraid (GB), a standardized assembly system based on type IIS restriction enzymes that allows the indefinite growth of reusable gene modules made of standardized DNA pieces. The GB system consists of a set of four destination plasmids (pDGBs) designed to incorporate multipartite assemblies made of standard DNA parts and to combine them binarily to build increasingly complex multigene constructs. The relative position of type IIS restriction sites inside pDGB vectors introduces a double loop (“braid”) topology in the cloning strategy that allows the indefinite growth of composite parts through the succession of iterative assembling steps, while the overall simplicity of the system is maintained. We propose the use of GoldenBraid as an assembly standard for Plant Synthetic Biology. For this purpose we have GB-adapted a set of binary plasmids for *A. tumefaciens-*mediated plant transformation. Fast GB-engineering of several multigene T-DNAs, including two alternative modules made of five reusable devices each, and comprising a total of 19 basic parts are also described.

## Introduction

Synthetic Biology adapts the general engineering principle of assembling standard components, dating back to the Industrial Revolution, to biological components. This discipline aims at the design of artificial living forms displaying new traits not existing in nature [1], [2]. This objective can be pursued following a bottom-up strategy, by creating new living forms from its basic components; however, a more straightforward option consists of integrating new genetic circuits within the genome of a current living organism or “chassis”. In this top-down tinkering approach, the construction of new versions of an existing organism can be conducted following a modular hierarchical approach, by combining well defined basic DNA “parts” (e.g. promoters, coding sequences, terminators, etc.) into genetic devices (e.g. transcriptional units), those devices into basic genetic modules (e.g. biochemical pathways, genetic circuits, etc.), and those into higher order modules, which integrated in a natural genome or “chassis” will configure a redesigned organism displaying new traits. Modularity is not only an engineering strategy; multiple high-throughput genetic interaction studies have provided substantial evidence of modularity in the genetic organization of cellular systems [3]. In view of this fundamental modular structure of genetic networks, many key design solutions are likely to involve intermediate hierarchical levels, entailing structures ranging from a few devices to complex modules and comprising between five and a few hundred basic genetic parts. In recent years the ability to manufacture synthetic DNA molecules has increased exponentially. Chemical synthesis ordinarily produces *de novo* sequences in the size range of a genetic “part” (up to 0.5–5 Kb) [4], [5]. On the opposite side, increasingly efficient homologous recombination methods have enormously facilitated the assembly of large DNA sequences up to the genome range [6], with the synthesis of a complete bacterial genome serving as best example [7], [8]. Despite these technical advances, many critical engineering issues as the exhaustive characterization of new genetic modules, their re-adaptation for additional purposes or their combination with other devices to produce combined traits still require from increasingly efficient and versatile DNA assembly methods operating at intermediate range. Moreover, to facilitate engineering at this level, basic pieces (parts) need to be assembled following standard rules, which can be applied independently of the identity of the parts. Standardization is therefore a crucial feature that allows the exchange of pieces among laboratories and facilitates automation. Standardization also favors reusability, as any standard pieces can be exchanged for assembling different constructs following common rules of assembly.

When adopting standardization, it is highly preferable that the rules of assembly are kept to a minimum. Simplicity facilitates the adoption of the technology by the potential users, reduces the elements in the engineer's tool box and simplifies the automation process. The maximum expression of simplicity in assembly standards is idempotency, occurring when any new composite part can be assembled following the same rules used to generate its original components. Idempotency is at the basis of the success of the BioBricks, a community effort to build a standardized collection of genetic parts for Synthetic Biology [9]. BioBricks standards are binary assembly rules where two pieces flanked by a set of restriction sites, result, upon assembly, in a composite piece flanked by identical restriction sites than their predecessors. The simplicity of the idempotency has boosted the interest in BioBricks standards, which have evolved to deal with engineering drawbacks as those derived from the presence of assembly scars [10].

BioBrick assemblies are strictly binary, meaning that only two elements can be assembled together in each assembly step. This feature slows down the engineering process, this being apparently an obligate penalty for idempotency. Oppositely, multipartite systems have been developed allowing the assembly of multiple DNA fragments in a single step. Among them, Golden Gate, a cloning system based on the use of Type IIS restriction enzymes, has a number of interesting features for operating at the level of genetic devices and modules [11], [12]. Unlike other multipartite methods, which are often based on overlapping flanks and *in vitro* recombination, Golden Gate cloning does not require PCR amplification of each part prior to the assembly. Since amplification of self-complementary or repetitive parts can be problematic, Golden Gate is more permissive than other methods for the assembly of repetitive elements. Despite being based on restriction/ligation, its all-in-one-tube design avoids inconvenient gel extraction procedures that often reduce cloning efficiency; most interestingly, it allows seamless assembly by careful design of the restriction sites. This feature is particularly important when DNA fragments comprise coding sequences for sensitive applications (e.g. in the design of therapeutic proteins). Despite its obvious advantages, Golden Gate, as many multipartite systems, is limited in standardization and reusability. Hence, Golden Gate multipartite assemblies, as originally designed, cannot be reused to generate higher order devices and modules following standardized rules of assembly, limiting its use in Synthetic Biology.

Here we present GoldenBraid, a new modular assembly system that allows the binary combination of multipartite assemblies using an extremely simple set of rules, very close to idempotency. GoldenBraid makes use of the multipartite Golden Gate cloning method to generate a modular assembly of standardized basic parts, which are then incorporated to a double loop (“braid”) cloning design that allows binary assembly of multipartite constructs. In this way, GoldenBraid technology enables the standardization of Golden Gate for its use in Synthetic Biology. Moreover, this is achieved with a small toolbox consisting of only four destination plasmids and a limited number of assembly rules.

Multigene engineering has an enormous potential in crop design, as for metabolic engineering, biofortification, molecular farming or for combination of traits of agronomic value via gene stacking [13]. Plant Synthetic Biology is a nascent discipline where the use of standard assembly rules has not yet rooted, and there is therefore room for efficient and innovative assembly methods to be adopted by the plant research community. Based on the features of GoldenBraid, here we propose its adoption as a common assembly standard for Plant Synthetic Biology. To substantiate this proposal we show here three examples of GoldenBraid-assisted multigene engineering in plants. In a first example we demonstrate the advantages of *in-cis* multigenic designs for *Agrobacterium*-mediated transient co-transformation. In a second example, we show the versatility of the system to assay recombinant antibody expression in a combinatorial way. Finally, we combine different modules to produce two alternative 14.3 Kb constructs each involving the assembly of 19 basic parts grouped in five different transcriptional units.

## Results

### Part standardization and multipartite assembly of simple devices

**Figure 1:**
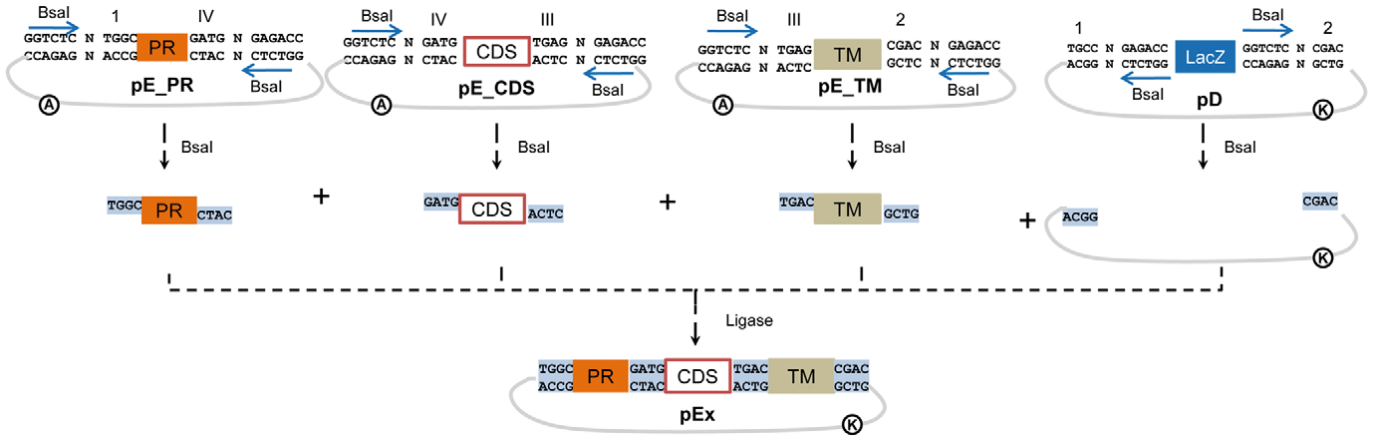
Part standardization and multipartite assembly of single devices. PCR products of entry plasmids (pE) containing basic parts such as promoters (PR), coding sequences (CDS) and terminators (TM) are flanked by fixed convergent BsaI recognition-cleavage sites. To facilitate the visualization of the design, we assigned each 4 bp cleavage sequence a different label: those produced by BsaI digestion are labeled with Arabic and Latin numbers (1,2,3, III, IV, etc). In assembling a single device, constituent parts (pEs) are incubated together with a destination plasmid (pD) containing a LacZ cassette flanked by BsaI sites in divergent orientation. As a result, an expression plasmid (pEx) is created where all BsaI recognition sites have disappeared**.** Encircled letters represent antibiotic resistance genes: A for AmpR, and K for KanR.

GoldenBraid is an adaptation of Golden Gate to Synthetic Biology. Golden Gate is a multipartite assembly system based on the use of type IIS restriction enzymes. These enzymes digest DNA at a defined distance few nucleotides away from its recognition site, not requiring any specific sequence in the actual cleavage site, and often leaving a short overhang. This feature makes them extremely useful in seamless cloning strategies: by carefully positioning recognition and digestion sites in opposite directions in entry and destination vectors, it is possible to design and obtain multipartite assemblies where all recognition sites in the final expression vectors have disappeared. Since there are no sequence requirements in the cleavage sites, these can be user-defined, and therefore accommodated to serve as assembly boundaries for standard DNA parts. Following this rationale, we initially considered three categories of basic parts, namely promoters (PR), coding sequences (CDS) and terminators (TM). All parts are cloned as BsaI fragments in entry clones. The inclusion in a category is defined by the flanking BsaI digestion sites. A schematic view of a standardized multipartite assembly of a transcriptional unit is depicted in Fig 1. To facilitate the interpretation, we gave a label to each 4 bp cleavage site producing the corresponding overhang (e.g. numbers 1, 2, 3, IV, etc, to those sites digested by BsaI enzyme). Therefore a promoter is a “part” flanked by sites 1 and IV, whereas CDSs are flanked by sites IV and III, and terminators are flanked by sites III and 2. In our approach, nucleotide boundaries were conveniently fixed to accommodate the nature/sequence of the different parts: site IV, defining PR-CDS boundary, was designed GATG, conveniently containing an ATG start codon, whereas site III, that forms CDS-TM boundary was designed to contain a TGA stop codon (namely TGAG). Parts are ordinarily created by PCR amplification of suitable templates, adding appropriate BsaI extensions to the primers. Once amplified, parts can be used directly as PCR fragments and/or cloned and stored in a collection for future assemblies.

### The Double loop Design of the GoldenBraid system

So far, the described method allows standardization, but the resulting units (expression vectors), lacking restriction sites, cannot be re-used in subsequent assembly reactions. A possible solution to this constraint would be the addition of restriction sites for a second type IIS enzyme (e.g. BsmBI) in the backbone of the destination plasmid, so that BsaI-assembled devices (first order assembly) could similarly be assembled in second order destination plasmids. However, in order to allow multipartite second order assemblies, this solution would require the design of a large number of destination plasmids, as the flanking BsmBI sites of the destination plasmids need to be different depending on the number of elements to be assembled in the second level. Moreover, in order to make the resulting composite parts fully reusable, an indefinite number of additional destination plasmids for subsequent hierarchy levels would be required.

**Figure 2:**
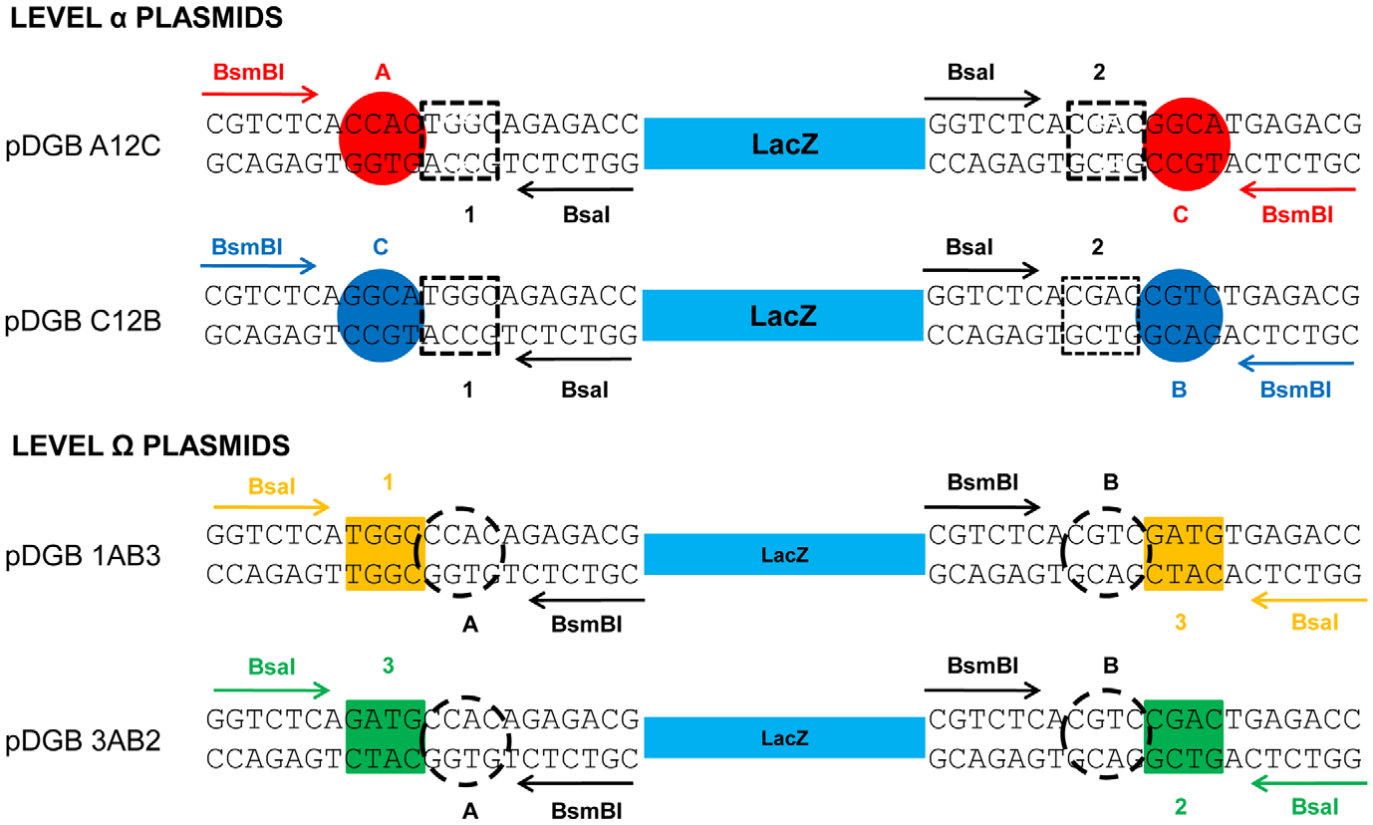
Structure of the LacZ cassettes in the GoldenBraid system. GB plasmid set comprises four destination plasmids (pDGBs), two of them act as destination plasmids for level α assembly and the remaining two function as destination plasmids for level Ω. All pDGB vectors incorporate a LacZ selection cassette flanked by four Type IIs restriction sites (BsaI, BsmBI), but positioned in inverted positions and orientations. To facilitate the visualization of the design, we assigned each 4 bp cleavage sequence a different label: those produced by BsaI digestion are labeled with squares and named with Arabic numbers (1,2,3), whereas BsmBI 4 bp cleavage sites are encircled and named with capital letters (A,B,C).

A simple solution to this limitation, described here as GoldenBraid, is to insert a loop (braid) in the cloning design, so that the expression plasmids from first level become entry plasmids for second level assemblies and vice versa. In order to do this, two types of destination plasmids were designed, namely level α and level Ω. The key in GoldenBraid design is that, while all plasmids contain two restriction/recognition sites corresponding to two different type IIS enzymes, level α and level Ω plasmids are designed to have their sites in inverted orientations (Fig 2). They also differ in the resistance marker associated to each of them, allowing counterselection. According to this strategy, only four destination plasmids are required to conform the loop cloning topology of GoldenBraid: plasmids pDGB_A12C and pDGB_C12B for assembling at level α and pDGB_1AB3 and pDGB_3AB2 for assembling at level Ω, where 1, 2 and 3 correspond to sequences of four nucleotide-overhangs produced by BsaI and A, B and C refer to the four nucleotide-overhangs produced by BsmBI.

**Figure 3:**
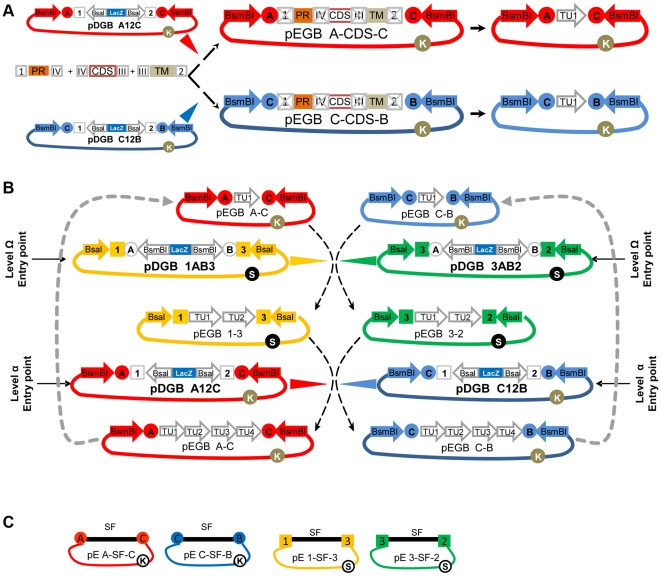
The mechanism of GoldenBraid system. (A) Standard parts as promoters (PR), coding sequences (CDS) and terminators (TM), flanked by fixed BsaI cleavage sites (represented as Arabic and Latin boxed numbers) are ordinarily assembled using level α plasmids (pDGBA12C or pDGBC12B). As a result of multipartite assembly, BsaI recognition sites disappear and the resulting boundary is not cleavable anymore (represented as a crossed label). Nevertheless, the newly assembled transcriptional unit (TU1, represented for simplification as an arrow) remains flanked by BsmBI cleavable sites (represented as encircled capital letters). (B) Two transcriptional units assembled in complementary α plasmids can be reused as entry vectors (pEGB) for a subsequent level Ω binary assembly, provided that they share a BsmBI sticky end (labeled as encircled C). Similarly, constructs assembled using opposite Ω plasmids can be reused as entry vectors for a subsequent level α binary assembly, provided that they share a BsaI sticky end (labeled as squared 3). Level α and level Ω can alternate indefinitely creating increasingly complex structures, as depicted by the arrows closing the double loop. Encircled K and S represent KanR and SpecR respectively. (C) Representation of the four “twister” plasmids that can be eventually used to assist GoldenBraid cloning design. SF is a 150 bp stuffer fragment containing an intergenic region from *Solanum.*

The cloning methodology used in GoldenBraid is shown in Fig 3. Standard parts are normally assembled in level α plasmids (Fig 3A). Those composite parts built into pDGB_A12C as destination vector can be merged with other structures assembled in pDGB_C12B, yielding two possible results depending on which of the two level-Ω plasmids is used as destination vector: a new structure flanked by 1–3 sites and/or a structure flanked by 3–2 sites (Fig 3B). In a second assembly round, composite parts assembled using level Ω plasmid can be assembled together using level α destination plasmids. As can be observed in Fig 3, GoldenBraid works as endless iteration of binary assemblies where the only limitations would be those imposed by the host on the size/composition of the DNA that can be stably propagated in a given destination vector backbone.

GoldenBraid assembly can be formally described with a simple system of four assembly rules:

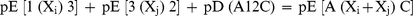

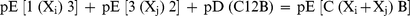

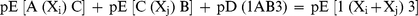

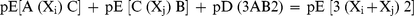

where,

- (X_i_) and (X_j_) are any DNA pieces, including Golden Gate assembled composite parts.
- (X_i_+X_j_) is a composite part of (X_i_) and (X_j_) that follows the same assembly rules than (X_i_) and (X_j_).
- Numbers 1, 2, and 3 are four-nucleotide sequences, which flank (X) pieces, and which are made protuberant ends upon BsaI digestion.
- Letters A, B and C are four-nucleotide sequences, which flank (X) pieces, and which are made protuberant ends upon BsmBI digestion.
- pE[ ] is any plasmid (entry plasmid) hosting a piece (X), such piece flanked by sites as indicated by flanking numbers or letters.
- pD( ) is any plasmid (destination plasmid) hosting a LacZ cassette, such lacZ cassette flanked by two sites, as indicated by flanking numbers or letters.

As deduced from these rules, in order to be GB-assembled together each DNA fragment needs to be cloned in a different plasmid from the same GB level. A careful design of the assembly strategy will ensure in most cases that two pieces to be assembled are correctly positioned. For those cases where this is not possible (e.g. two devices designed independently in different labs), we have constructed four “twister” plasmids containing a small stuffer fragment that facilitate moving pieces from one level to the next in a single GB reaction (Fig 3C). The twister plasmids are indeed four entry plasmids hosting a “fixed” tomato intergenic region flanked by one of the four possible enzyme combinations each (A–C, C–B, 1–3 or 3–2). Using these plasmids, any GB-cloned composite part can be easily and conveniently GB-twisted into next level plasmids, allowing its assembly with parts located at the opposite level.

It is highly desirable that all the components in the GoldenBraid system are free of internal BsaI and BsmBI sites. For part domestication, internal sites are removed using standard methodology as overlapping-PCR, directed mutagenesis, or direct DNA synthesis. For plasmid adaptation to GB system, we followed a general procedure using a third type IIS enzyme (BbsI). The original binary plasmid was deconstructed in pieces; the number of pieces depends on the number of internal sites to be removed and the functional structures that need to be kept as independent pieces. Usually, basic pieces involve the lacZ cassette, antibiotic resistance, and two additional pieces containing replication origins and each of the T-DNA borders. Four lacZ pieces (A12C, C12B, 1AB3 and 3AB2) and two different antibiotic resistance pieces (e.g. KanR and SpmR) are to be produced to generate a complete GB plasmid set. Additional pieces may be required to mutagenize internal type IIS sites.

### Multigenic constructs for Plant Biology

#### GoldenBraid-assisted co-transformation ensures the coordinated expression of multiple genes in transient expression experiments

*Agrobacterium*-mediated transient gene expression (agroinfiltration) in *Nicotiana benthamiana* is an efficient technology for recombinant protein production in plants. An interesting feature of this system is the high co-transformation efficiency obtained by simply combining two or more independent *Agrobacterium* cultures each carrying one of the genes of interests (this called *in trans* co-transformation). The cumbersome and inefficient assembly of multiple transcriptional units in a single T-DNA has often led many labs to rely on *in trans* co-transformation when the coordinated or simultaneous expression of two or more proteins in a single cell/tissue was pursued. The GoldenBraid strategy here described makes the cloning of multigene constructs a straightforward task. To test whether an *in cis* co-transformation approach outperforms the *in trans* approach, three different fluorescent devices were GB-assembled and its performance compared with that of an *in trans* approach.

**Figure 4:**
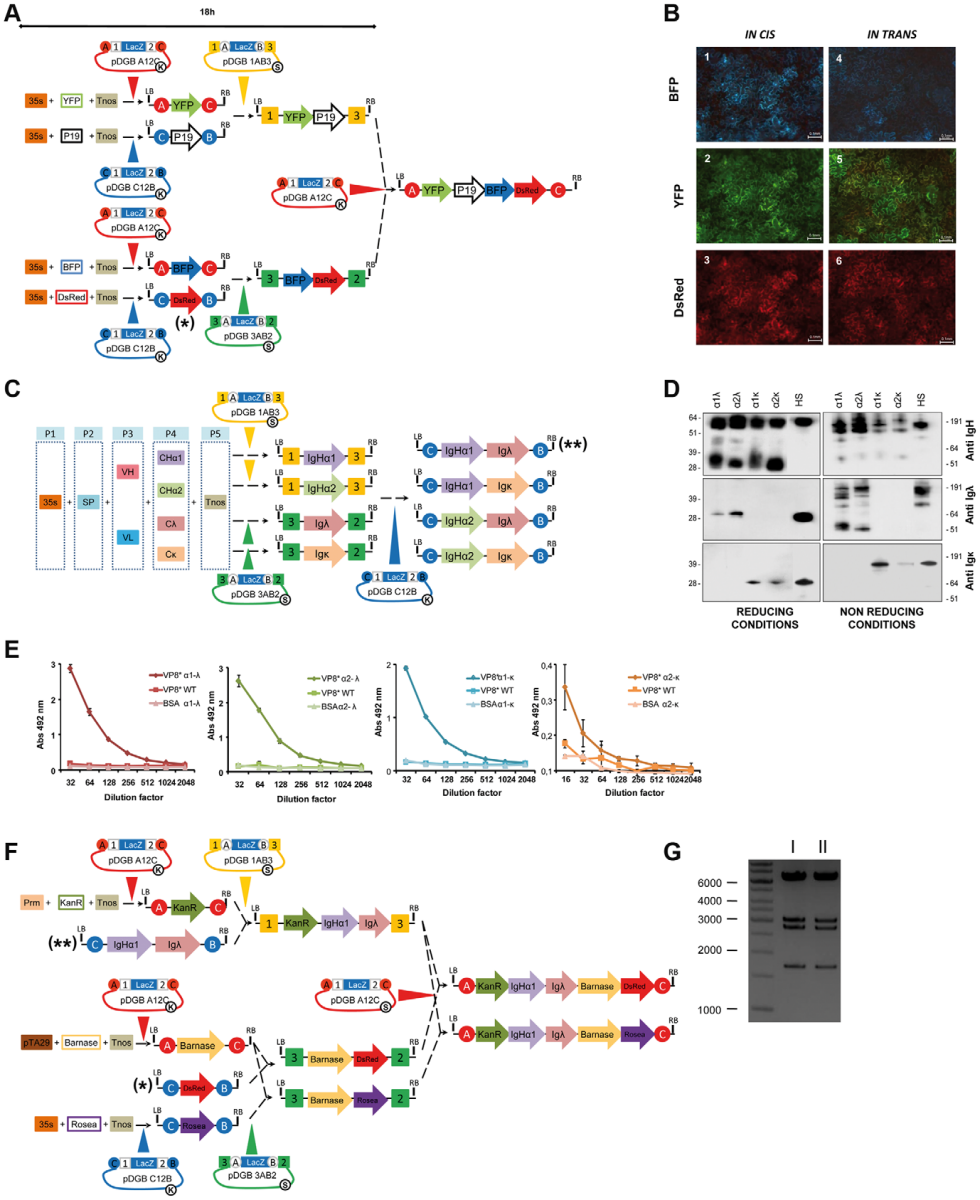
Multigenic constructs for plant biology. (A) GoldenBraid cloning path for the assembling of YFP, p19, BFP and DsRED transcriptional units in a single T-DNA. (B) Spatial expression patterns of BFP, YFP and DsRed in *N. benthamiana* leaves agroinfiltrated with pEGB_A-YFP-P19-BFP-DsRed-C- (left captures, 1, 2 and 3) or with a mixture of the individual devices pEGB_A-YFP-C, pEGB_C-p19-B, pEGB_A-BFP-C and pEGB_C-DsRed-B (right captures 4, 5 and 6). (C) GoldenBraid cloning strategy followed in the assembly of different IgA isotypes. Multipartite assembly involved the combination of different basic parts each occupying a fixed position in the assembly (P1-P5). Individual antibody chains were assembled in pDGB_C12B plasmid to yield four IgA isotypes. 35S is CaMV35S promoter; SP, pectate lyase signal peptide; CHα1 and CHα2, are heavy chain constant domains; Tnos, is nopaline synthase terminator; Cλ and Cκ, are light chain constant domains; VH and VL are heavy and light variable regions of an antibody against rotavirus VP8* peptide. Promoter and terminator pieces were flanked by the same 4 nucleotide extensions as in Fig 1. Signal peptides incorporated a GATG extension at its 5′ end, whereas constant antibody regions ended in TGAG extensions to match terminators. The remaining boundaries were designed to produce benign junctions within coding sequences. (D) Western Blot analysis of IgA transient expression in *Nicotiana benthamiana*. Leaves were infiltrated with the four previous combinations. Samples were resolved under either reducing (left) or non-reducing (right) conditions and decorated using anti-heavy chain antibody, anti- λ light chain antibody or anti- κ light chain antibody. HS lane contains control human serum. (E) End-point antigen-ELISA tittering of four IgA combinations tested by transient expression in *Nicotiana benthamiana* leaves. All samples were tittered against VP8* or against BSA and compared with equivalent samples derived from wild type leaves (WT). (F) GoldenBraid strategy for the assembly of two alternative 5-gene T-DNA constructs. (G) PvuI digestion of one colony of each final constructs pDGB_A-KanR-IgHα1-Igλ-Barnase-Rosea-C (lane I) and pDGB_A-KanR-IgHα1-Igλ-Barnase-DsRed-C (lane II). Asterisks highlight those GB-assembled transcriptional units that were reused in the assembly of new multigenic structures.

As starting point for the assembly, we used a small collection of basic parts (pEs), namely promoters, CDS and terminators. Fluorescent devices (transcriptional units) were BsaI-assembled into GoldenBraid Level α vectors (Fig 4A). Three basic parts were assembled in each case: pE_35S (35S promoter) and pE_Tnos (nopaline synthase terminator) were used in all the constructions and assembled to CDS parts carrying either a yellow fluorescent protein (pE_YFP), a blue fluorescent protein (pE_BFP), a TBSV silencing suppressor (pE_p19) or *Discosoma* sp. red fluorescent protein (pE_DsRed) respectively. Two of the resulting devices (YFP and BFP transcriptional units) were assembled into pDGB_A12C and the two others (DsRed and p19 transcriptional units) were assembled into pDGB_C12B, generating four expression vectors: pEGB_A-YFP-C, pEGB_A-BFP-C, pEGB_C-p19-B and pEGB_C-DsRed-B. These reactions were extremely efficient with an average of 64000 colonies obtained in each transformation (generally 4 colonies were selected for mini-prep resulting in 100% correct colonies). Next, pEGB_A-YFP-C and pEGB_C-p19-B were assembled together into pDGB_1AB3, whereas pEGB_A-BFP-C and pEGB_C-DsRed-B were assembled into pDGB_3AB2, generating the expression vectors pEGB_1-YFP-p19-3 and pEGB_3-BFP-DsRed-2 respectively with the same high efficiency and accuracy.

Taking advantage of the different selection markers of the plasmids in levels α and Ω, we also tested the possibility of building double-device constructs directly from its basic parts in a single *in vitro* experiment. Ordinarily, devices are BsaI-assembled in one-tube multipartite reactions using α level destination plasmids, and the resulting mix is used to transform *E. coli*. In this case, the double-device constructs were attempted by combining two independent single-device reactions (e.g. pEGB_A-BFP-C and pEGB_C-DsRed-B) in a new tube and incubating with BsmBI and ligase for additional 25 cycles. As a result the two functional devices were assembled in one T-DNA (pEGB_3-BFP-DsRed-2) with 1/10 efficiency of the two-step assembly, but in a single day experiment and without requiring intermediate *E.coli* transformation.

Finally, pEGB_1-YFP-p19-3 and pEGB_3-BFP-DsRed-2 vectors were assembled in a BsaI reaction into the destination vector pDGB_A12C. This final multigenic construction pEGB_A-YFP-P19-BFP-DsRed-C, comprising 11.4 Kb and 12 parts, was functionally validated by agroinfiltration into *N. benthamiana* leaves. In parallel, single-assembled fluorescent proteins and p19 were also co-transformed *in trans* by mixing their respective *Agrobacterium* cultures. As can be observed in Fig 4B, GoldenBraid assembled fluorescent proteins showed coordinated expression in *N. benthamiana,* as deduced by the similar fluorescence intensity observed in all three channels. In contrast, when the fluorescent devices were agroinfiltrated *in trans*, each channel showed a different intensity distribution, evidencing heterogeneous expression levels of the different proteins.

#### GoldenBraid-assisted antibody chain shuffling facilitates selection of antibody isotype

The plant-based production of therapeutic antibodies is a field that requires flexible multigene cloning strategies. Therefore we evaluated our GoldenBraid-assisted cloning to build different “antibody devices” and compared the results obtained after expressing the proteins *in planta*. In the previous experiment with fluorescent proteins, parts were BsaI-assembled into level α plasmids (entry point α in Fig 3). The loop design of GoldenBraid system should allow the use of both level α and level Ω plasmids for multipartite assembly of basic parts. In this second experiment we made use of entry point Ω to build and assemble basic parts for therapeutic devices. A versatile strategy was designed to assemble any desired human IgA (h_IgA) isotype. To gain flexibility, parts were classified in five categories, namely promoter, signal peptide, variable antibody regions, constant antibody regions and terminators. Next, five-part BsmBI reactions were performed to assemble the individual heavy and light antibody chains. The experiment showed here was aimed at selecting the best IgA isotype for in planta production of an anti-rotavirus antibody. For this purpose, two heavy chains (pEGB_1-IgHα1-3 and pEGB_1-IgHα2-3) and two light chains (pEGB_3-IgK-2 and pEGB_3-Igλ-2) were BsmBI-assembled into level Ω plasmids. Next, heavy and light chain devices were combined in a BsaI-GoldenBraid reaction, generating the four different isotypes of human IgA (Fig 4C). The four h_IgA isotypes produced (separately) in agroinfiltrated leaves were compared by western blot (Fig 4D) and ELISA (Fig 4E), with the version combining IgHα1 and Igλ (pEGB_C-IgHα1-Igλ-B) showing best performance *in planta*.

#### Construction and combination of therapeutic and biosafety gene modules by GoldenBraid

One of the strengths of GoldenBraid cloning is the reusability of pieces, so once assembled and tested for one purpose they can be easily included in further multigenic structures aimed at similar or different purposes. To illustrate this ability, we show the use of some of the devices described above to make two additional multigenic structures (Fig 4F). In this case a “therapeutic” module (anti-rotavirus IgA) initially aimed at transient expression is reused for a different purpose, the engineering of a biosafe plant biofactory for anti-rotavirus IgA. For this goal, IgA “therapeutic” module is combined with a “selection” device for plant stable transformation (KanR) and two alternative biosafety modules, both comprising an “identity preservation” device and a “polen-sterility” device.

Single-device constructs were assembled as follows (Fig 4F): first a Kanamycin resistance device was built in a multipartite BsaI reaction into level α plasmid pDGB_A12C. Next, two alternative “Identity Preservation” devices were considered: the previously described pEGB_C-DsRed-B conferring red fluorescence to the plant, and the newly constructed *Rosea1*, consisting of a 35S, Nos terminator and the *Antirrhinum majus* Rosea1 transcription factor that confers purple color to the cells [14]. Finally a male sterility “device” was constructed, combining barnase-barstar CDS under pTA29 anther-specific promoter [15], [16]. From here, the assembling of multigene structures was conducted as follows: the device pEGB_A-KanR-C was assembled to the IgA “therapeutic” module in a BsmBI reaction into pDGB_1AB3. Next, two alternative “biosafety” modules, namely pEGB_3-Barnase-Rosea-2 and pEGB_3-Barnase-DsRed-2 were assembled into level Ω plasmids as shown in Fig 4F. Finally, biosafety modules were assembled to the IgA_KanR module in a final BsaI reaction resulting in two alternative five-device constructs of 14.3 Kb and 19 pieces made of reused devices (Fig 4G).

## Discussion

GoldenBraid is a tool that converts single-use Golden Gate multipartite assemblies into reusable composite parts. In this sense GoldenBraid assembly is an attempt to extend the capabilities of the previously described Golden Gate cloning system to the requirements of Synthetic Biology. There are no preconditions on the type of DNA pieces involved in the initial multipartite assembly, which can be either basic parts, transcriptional units or even small pathways. However, we think that multipartite assemblies of basic DNA parts are most interesting, particularly when this is made in a standardized, community-based fashion. To do so, we propose (i) the creation of a standardized collection of basic parts flanked by type IIS sites, (ii) the multipartite assembly of DNA parts into GB destination plasmids to generate simple genetic devices; (ii) the use of GB plasmids and GB rules to grow increasingly complex genetic modules and pathways.

Part standardization is pivotal for genetic engineering. The small junctions used by type IIS-based cloning and the high efficiency of GoldenBraid procedure greatly favors standardization. We currently use a small collection of basic parts structured in promoters, CDS, and terminators, however, a more elaborated category list could be considered. It is important to notice that the relative position of a DNA fragment in a multipartite assembly, and therefore its identity, is determined by its 4-nucleotide flanking sequences. Adoption of common sequences by different labs would be required for taking full advantage of the system.

We think GoldenBraid has a number of characteristics that encourage its adoption by scientific community. One of them is **reusability/exchangeability**: all GoldenBraid composite parts can be either transformed directly into cells or used as a piece to build more complex structures. No PCR amplification or further modifications of the piece are required. Error-born and/or lengthy adaptation methodologies hamper the engineering processes, whereas full reusability ensures the reproducibility of the built-in genetic devices. A second advantage is **speed**: as the starting point of GoldenBraid scheme is a multipartite assembly, the overall engineering process is considerably accelerated when compared with purely binary systems as Biobricks. Moreover, we have shown that two expression cassettes can be assembled together in less than 24h starting from basic parts. A third comparative advantage is **accuracy**: Type IIS cloning allows the building of assemblies containing short “benign” seams, as earlier demonstrated in Golden Gate cloning. Finally, a distinctive characteristic of the GoldenBraid scheme is its **simplicity**: GoldenBraid can theoretically build indefinite assemblies with the only use of four destination plasmids and four basic assembling rules.

Plant genetic engineering currently relies on assembly methodologies poorly adaptable to Synthetic Biology. In an attempt to facilitate versatile cloning into plant binary vectors, we and others have developed plasmid collections based on Gateway technology [17], [18], [19]. Gateway cloning, based on site-specific recombination, is a highly efficient cloning technique; however it leaves long scars between pieces (attB sites) and the reusability of pieces is limited. A number of additional techniques, based on site-specific recombination, the use of rare cutters or homing endonucleases have been developed [20]-[24], however in our opinion GoldenBraid compares favorably with most of them in terms of standardization, simplicity and reusability.

In view of this need, we have adapted GoldenBraid scheme to plant biotechnology by domesticating four binary plasmids, and demonstrated in a number of examples the feasibility of the methodology. In a first example, using fluorescent proteins, it was demonstrated that GoldenBraid is permissive with the repetition of single pieces in multiple assemblies. At least as long as transient expression is concern, the introduction of 4 copies of 35S promoter in a single T-DNA does not affect the transient expression of the fluorescent proteins. Just on the contrary, *in cis* co-transformation favors the coordinated expression of the transgenes. *In trans* co-agroinfiltration is currently used as a fast–track tool for e.g. plant glyco-engineering or metabolic engineering, both approaches often relying on coordinated expression of the different transgenes in each cell [25]. In the light of the results showed here, GB-assisted assembling would improve the outcome of these transient approaches, as it would do so if the same engineered T-DNAs were to be stably transformed in plants.

In a second example we illustrate the use of GB in antibody engineering by exchanging in a combinatorial way all the alternative constant regions of a human IgA against rotavirus. Moreover, this design also allows the exchange of variable regions, facilitating conversion of antibody idiotype. In this particular example we chose to build parts that enter the GB loop at the Ω level, therefore demonstrating the symmetry of the braid. Although this possibility remains open, it seems more reasonable for a general strategy the use a single entry level, as this facilitates part standardization. It is important to notice that, in its current design, GB uses different entry sequences for level α (sites 1 and 2) and level Ω (sites A and B). It could be conceived a system where A = 1 and B = 2, which would allow standard pieces to be assembled indistinctly at level α or Ω. This would increase the exchangeability of the pieces, reducing the eventual need for twister plasmids. However, this would also require the use of an additional type IIS restriction enzyme for the cloning of basic “parts”. By doing so, parts could be multi-partite assembled at any level by using an “extra” enzyme that does not destroy the restriction sites to be used at the next level. In this case, the increased reusability would pay the toll of extra domestication requirements introduced by a third enzyme. We calculate that, by using our current two-enzyme design, 29% of tomato cDNAs would require domestication, whereas the use of a third enzyme (e.g. BbsI) would increase this figure up to 51%. Considering the simplicity and efficiency of helper-assisted twists, we tend to favor current design over a three-enzyme design.

In a final example we demonstrate the reusability of GB constructs with the assembly of two alternative constructs comprising five transcriptional units. A “therapeutic” module (IgA) is combined with a “selection” module and two alternative “biosafety” modules. Biosafety modules are made of a “male sterility” device and two alternative “identity preservation” devices. In our opinion, this example fully illustrates the principles of modularity, standardization and reusability that drive Synthetic Biology aims.

Given the indefinite design of GB, the obvious limitation to GB assemblies is that imposed by the maximum insert size that can be harbored by binary plasmids. Although initially designed using binary plasmids, GB assemblies, as fully reusable units, can be easily transferred to newly domesticated structures such as BiBACs [26] suitable to host larger T-DNAS, or other devices for direct DNA transfer. Moreover, at any time GB constructs can be added new pieces that facilitate its conversion to alternative assembling methods. This may include, among other elements, attB cassettes for Gateway cloning, overlapping regions for in vitro or in vivo recombination, or recombination sites (e.g. loxP) for *in planta* gene stacking. We consider that standardized *in vitro* gene assembling methods as GB may become an important tool in engineering of complex traits, which lays at the horizon of modern Plant Biotechnology.

During the preparation of this manuscript, an alternative methodology for the standardization of Golden Gate cloning for Synthetic Biology (named MoClo) was published [27]. In their paper, Weber *et al*. show the construction of a 33 Kb multigenic structure with the only use of successive Golden Gate reactions, a result that demonstrates that type IIS technologies (including GoldenBraid) can successfully be used for the assembly of complex genetic modules. MoClo proposes an elegant strategy for the cloning of “subparts” (level 0) that was not contemplated in GB strategy. This interesting strategy enhances the flexibility and the combinatorial power of any part collection. Also, similarly to GB, MoClo proposes the use of a second enzyme in destination plasmids as a way to extend Golden Gate cloning to a second assembly level. The use of a second enzyme for extended cloning has been also very recently proposed by different authors as a tool to facilitate modular assembling of TAL effectors [28]–[31], however MoClo brings this idea to a general scheme for multigene assembling. In MoClo strategy a first enzyme (BsaI) is used to assemble “parts” into devices (level 1, equivalent to GB level α), and a second enzyme (BbsI) is used to combine devices into multigene structures (level 2, equivalent to GB level Ω). However at this point the solutions provided by MoClo and GB to achieve the indefinite growth of multigene structures become completely different. As the use of two enzymes limits the level of successive assembling levels to two, MoClo proposes the creation of intermediate assembly levels (2i-1, 2i-2, etc), where an “extra” piece (end-linker) consisting of a selection cassette (LacZ or Red) is introduced as a way to leave the assembly “open” to the addition of new pieces. Further additions will involve the exchange of lacZ and Red cassettes by new “true” pieces in successive assembly levels.

**Figure 5:**
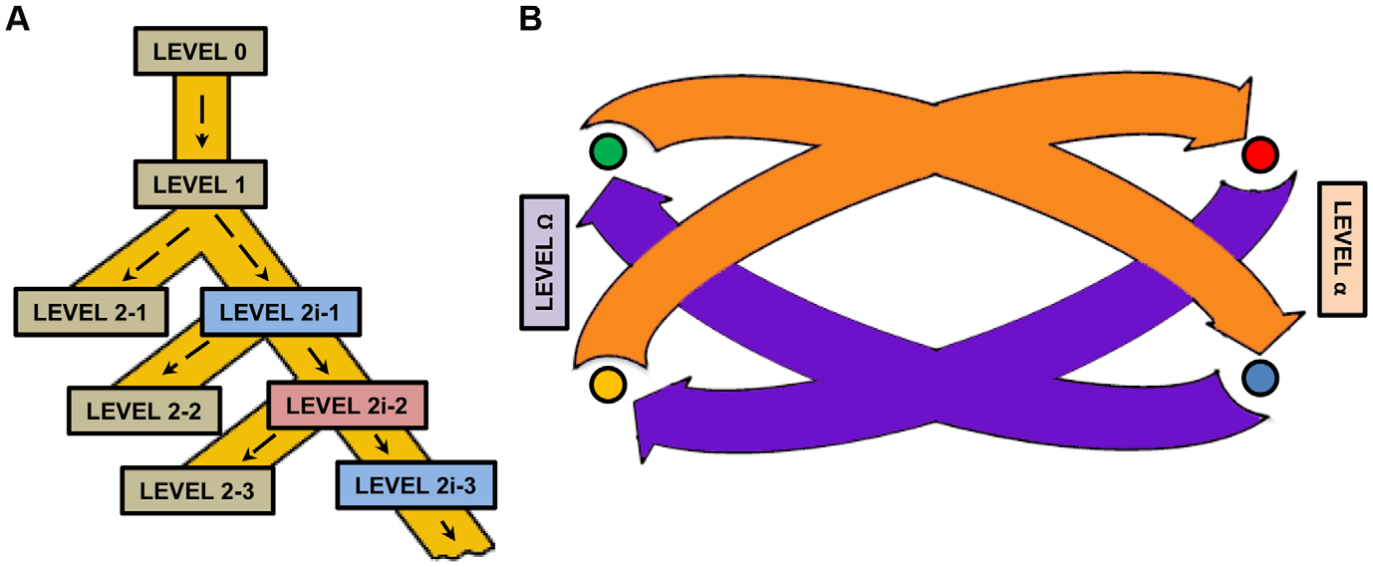
Comparison of the topology of MoClo and GoldenBraid. (A) Hierarchical topology of MoClo assembly. Level 0 hosts the flexible assembly of subparts into basic parts, allowing also part domestication. Level 1 hosts multipartite assembly of basic parts into transcriptional units. Level 2-1 hosts multipartite assembly of transcriptional units, yielding a non-reusable structure. Alternatively, level 1 can be branched into level 2-1i (intermediate) by adding an end-linker, yielding an open structure (albeit non functional), which can host new transcriptional units (level 2-2). Successive intermediate levels ensure the indefinite structure of the cloning system. (B) Double loop topology of GoldenBraid. Level-α plasmids host the multipartite assembly of basic parts into transcriptional units. Two level-α transcriptional units can be assembled together yielding two alternative level-Ω constructs, which themselves can be assembled into level-α constructs. The overall structure is a double iterative loop that ensures the indefinite growth of the assembly system.

GB has a number of features that differentiate it from the solution proposed by Weber et *al.*: (i) GB makes use of only two restriction enzymes whereas MoClo requires a third enzyme and an additional selection cassette to ensure indefinite growth; (ii) GB pieces are fully reusable, whereas in MoClo intermediate structures need to be assembled to allow further growth of the construct; (iii) GB assemblies are always binary, whereas MoClo allows multipartite assemblies at level 2; (iv) the topology of MoClo system is basically lineal, with successive assembly levels and lateral branches corresponding to intermediate levels. In contrast, GB has a circular topology, with pieces growing by alternating level α and Ω. A comparison of the topology of the two systems can be observed in Fig 5.

In synthesis, we consider that GB has two main distinctive features that can make it a useful alternative to MoClo for certain applications: its simplicity and the reusability of its composite parts. Conversely, MoClo main advantage is the possibility of building multipartite assembles at level 2. Both groups of features are probably mutually exclusive: MoClo multipartite assemblies at level 2 come at the expenses of the incorporation of a number of additional destination plasmids and end-linker plasmids to the system, which further increases its complexity. Analogously, additional destination and end-linker plasmids could be added to GB level α to allow multipartite assemblies at level Ω (e.g. A12D, D12C and C12B to obtain tripartite assemblies). However we doubt that the possible advances in speed could compensate the increased complexity of this solution provided that (i) indefinite growth of GB assemblies is ensured without the use of additional elements, (ii) intermediate binary assemblies are in itself useful as reusable entities (see last example of results section); (iii) in our experience multipartite cloning of large fragments has low efficiency, making often advisable to advance large constructs in binary form; (iv) speed in GB is satisfactory, as we show that 2-device assemblies can be constructed from its basic parts in a single *in vitro* 18h experiment; (v) the adoption of the technology by the community as well as its automation will be facilitated if simplicity is maintained.

It needs to be pointed out that both MoClo and GB are based on the same enzymatic reactions, and therefore, it can be expected that both should perform similarly in terms of construct size. The ability to assemble complex constructs will most likely depend on other factors not covered in this paper as the host plasmid (copy number, replication origin), the presence of repetitive regions, the host bacteria (whether *Agrobacterium*-mediated transformation is needed), etc. Either as GB or as MoClo, the extension of Golden Gate method to the standardized assembly of higher order genetic pieces as devices and pathways is an important step that will facilitate genetic engineering, particularly in the plant field. In our opinion, it would be highly beneficial to establish community-shared standards in aspects as piece identity and entry sites in order to facilitate the exchange of genetic pieces between labs and to facilitate further development of Plant Synthetic Biology.

## Materials and Methods

### Strains and growth conditions

*Escherichia coli* DH5α was used for gene cloning and *Agrobacterium tumefaciens* strain GV3101 was used for plant agroinfiltration and transformation experiments. Both strains were grown in LB medium under agitation (200 rpm) at 37°C and 28°C respectively. Ampicillin, kanamycin and spectinomycin were used for *E. coli* at 50 µg ml^-1^. Rifampicin, tetracycline and gentamicin were also used for *A.tumefaciens* at 50, 12.5 and 30 µg ml^−1^ respectively.

### Cloning and assembly of modular pieces

PCR amplification was performed by using the Advantage® 2 DNA Polymerase Mix (Clontech, California, USA) following the manufacturer's instructions. PCR was analyzed by agarose 1% gel electrophoresis and purified using the QIAquick PCR Purification Kit (Qiagen, Hilden, Germany). Amplified parts were TA Cloned using the pGEM®-T Easy Vector System (Promega, Madison, USA) and 1 µl of the ligation was transformed into DH5α electrocompetent cells. Plasmid DNA preparations were obtained by using The E.Z.N.A. Plasmid Mini Kit I (Omega Bio-Tek, Norcross, USA). Plasmid DNA concentration was measured using a Nano Drop Spectrophotometer 2000 (Thermo Scientific, Rockford, USA). Positive clones were selected in ampicillin-containing plates and confirmed by plasmid restriction analysis (EcoRI, NotI) and by sequencing.

Assembly reactions were performed basically as described by Engler et al. [11] using BsaI, BsmBI and BbsI as restriction enzymes in 25 cycle digestion/ligation reactions. Restriction enzymes were purchased from New England Biolabs (Ipswich, USA). T4 DNA ligase was purchased from Promega. One µl of the reaction was transformed into DH5α electrocompetent cells. Positive clones were selected in kanamycin or spectinomycin-containing plates. Plasmid DNA preparations were made by using The E.Z.N.A. Plasmid Mini Kit I (Omega Bio-Tek). Plasmid DNA concentration was measured using a Nano Drop Spectrophotometer 2000 (Thermo Scientific). Constructs were confirmed by plasmid restriction analysis and by sequencing. Constructs for plant functional assays were transferred to *Agrobacterium tumefaciens* electrocompetent strain GV3101.

### GB-Domestication of destination Plasmids for Plant Biology

With some adaptations, domestication of pDGB plasmids was performed basically as earlier described by Engler et al. [11]. A third type IIS enzyme was used (BbsI) for domestication. All the components in the GoldenBraid system were made free of internal BsaI and BsmBI sites. The original binary plasmid (pGreen II) [32] was deconstructed in four pieces involving the LacZ cassette, antibiotic resistance, and two additional pieces containing replication origins and each of the T-DNA borders. Four lacZ pieces (A12C, C12B, 1AB3 and 3AB2) and two different antibiotic resistance pieces (e.g. KanR and SpmR) were produced to generate a complete GB plasmid set. To assemble pDGB plasmids set, four BbsI Golden Gate reactions between backbone pieces and LacZ cassettes were set up, yielding the four pDGB plasmids, each containing a different LacZ cassette and the Kan or Spm resistance genes.

For the construction of twister plasmids, a small intergenic region (150 bp) was PCR-amplified from tomato gDNA, using BsaI and BsmBI primer extensions that match the cloning sites of each pDGB (i.e. 1–2 for BsaI and A-B for BsmBI). PCR fragments were purified and subsequently GB-cloned in each of the four destination plasmids.

### Plant transient transformation

For transient plant transformations plasmids were transferred to *Agrobacterium tumefaciens* strain GV3101 by electroporation. Agroinfiltration was performed as previously described [33]. Briefly, overnight grown bacterial cultures were centrifuged and the pellets resuspended in agroinfiltration medium (10 mM MES pH 5.6, 10 mM MgCl_2_, 200 µM acetosyringone) to an optical density at 600 nm  =  0.4. Co-infiltrations were performed by mixing equal volumes of the corresponding bacterial suspensions. Inoculations were carried out by syringe-agroinfiltration in leaves of 4–5 weeks old *Nicotiana benthamiana* plants (growing conditions: 24°C day/20°C night in a 16 h light/8 h dark cycle). Samples were collected 5–6 days post-infiltration and examined for transgene expression.

### Western Blot and ELISA Analysis

Detection of individual antibody chains and IgA complexes was carried out by western blotting. Leaf proteins were extracted in 3 volumes (v/w) of PBS (phosphate buffer saline, pH7.4). Protein separation was carried out by SDS-PAGE on NuPAGE 10% Bis-Tris polyacrylamide gels (Invitrogen, Paisley, UK). Proteins were transferred to PVDF membranes (Amersham Hybond-P, GE Healthcare, UK) by semi-wet blotting (XCell II^TM^ Blot Module, Invitrogen) following manufacturer instructions. Membranes were blocked with a 2% (w/v) solution of ECL Advance^TM^ Blocking agent (GE Healthcare, UK) in PBS-T (0.1% (v/v) Tween 20 in PBS). For the detection of IgH_α1 and α2 heavy chains membranes were incubated with 1∶20000 Anti-Human IgA (α-chain specific) peroxidase conjugate (SIGMA, St. Louis, USA); the Igλ and Igk light chains were detected by incubation with 1∶10000 Anti-Human lambda light chain (Sigma) and 1∶10000 anti-human-kappa chain (Pierce - Thermo Scientific) as primary antibodies, followed by an incubation with 1∶10000 ECL Rabbit IgG, HRP-Linked (GE Healthcare) and 1∶10000 Anti-Goat IgG-peroxidase (Sigma) respectively, as secondary antibodies. Blots were developed with ECL Plus Western Blotting Detection System (GE Healthcare) following manufacturer instructions and visualized by exposure to X-ray film (Fujifilm Coorporation, Tokyo, Japan).

The binding activity of the recombinant IgA was determined by ELISA. Plates (CORNING, New York, USA) were coated overnight with 10 ug/mL of recombinant VP8* in coating buffer (50 mM carbonate buffer pH 9,8) at 4°C. Plates were then washed 4 times in PBS and blocked with a 2% (w/v) solution of ECL Advance^TM^ Blocking agent (GE Healthcare) in PBS-T (0.1% (v/v) Tween 20 in PBS). Samples were diluted in PBS as required for each assay and incubated for 1 hour at room temperature. After incubation, plates were washed 4 times in PBS and the anti-human IgA α specific-HRP 1∶5000 (Sigma-Aldrich) in 5% blocking buffer (GE Healthcare) in PBS-T was added and incubated for 1 h at room temperature. After 4 PBS washes, the substrate (*o*-phenilenediamine from Sigma-Aldrich) was added and the reactions were stopped with 3M HCl. Absorbance was determined at 492 nm.
